# Optimization of Automatic Synthesis and Separation of [^18^F] AV-45 and Quality Control

**DOI:** 10.3389/fchem.2022.826678

**Published:** 2022-04-12

**Authors:** Qi-Zhou Zhang, Yu-Bin Li, Nazi Yilihamu, Xiao-Hong Li, Ya Ba, Yong-De Qin

**Affiliations:** Department of Nuclear Medicine, The First Affiliated Hospital of Xinjiang Medical University, Urumqi, China

**Keywords:** automatic radiochemical synthesis, [^18^F] AV-45, ^18^F^−^, HPLC purification, Alzheimer’s disease

## Abstract

**Objective:** Based on the Tracerlab FX_F-N_ platform, a synthesis program and preparative high-performance liquid chromatography (HPLC) purification program edited by us can stably and repeatedly produce [^18^F] AV-45 without changing the process. The [^18^F] AV-45 produced meets the main indexes of radiopharmaceutical intravenous preparations.

**Methods:** The O-toluene sulfonated precursor (1 mg) was subjected to nucleophilic radiofluorination at 115°C in anhydrous dimethyl sulfoxide (DMSO), then the protective group was hydrolyzed by acid. The neutralized reaction mixture was purified through a preparative HPLC then formulated for injection using a C18 purification cartridge. This method yielded a relatively pure [^18^F] AV-45 product with high specific activity.

**Results:** Four consecutive radiochemical synthesis operations were carried out in this experiment; the average production time of [^18^F] AV-45 preparation was 60 min, the radiochemical yield was 14.8 ± 2.1% (*n* = 4), the radiochemical purity was greater than 95%, and the other important quality control indexes met the requirements of radioactive drugs for intravenous administration.

**Conclusion:** This experiment was based on the Tracerlab FX_F-N_ platform with the synthesis program and preparative HPLC purification program edited by us. Through screening and optimization of the separation and purification system and the separation and analysis system, as well as automatic radiochemical synthesis and preparation quality control, intravenous [^18^F] AV-45 was successfully prepared.

## Introduction

Alzheimer’s disease (AD) is a chronic progressive mental decline disease with dementia as the main manifestation, particularly senile dementia. Statistics indicate that the incidence of dementia is 5% among people over 60 years old, increasing to 20% among people over 80. As mentioned in the 2018 report of World Alzheimer’s Disease, there will be one case of dementia in the world every 3 s; in 2018, about 50 million people worldwide suffered from dementia, and by 2050 this figure will increase to 152 million, three times the current value (Weidner and Barbarino, 2019). China is entering an ageing society, with around 15 million elderly people with dementia (Jia et al., 2020).

The pathogenesis of AD is extremely complex, including oxidative stress, synaptic abnormalities, etc.; the more recognized ones are the amyloid β-protein (Aβ) cascade hypothesis and the tau protein hypothesis for the formation of neurofibrillary tangles (NFT) (SantaCruz et al., 2005; de Paula et al., 2009; Di et al., 2016; Selkoe and Hardy, 2016; Levin and Vasenina, 2017). The main pathological features of AD are amyloid protein deposition and NFT in the brain. At present, the clinical diagnosis of AD is mainly based on clinical history and excluding other diseases, which depends on histopathological confirmation; these methods are not conducive to the early diagnosis of AD.

In recent years, neuromolecular imaging has developed rapidly. A number of teams have developed a range of promising positron emitting radioactive diagnostic drugs. [^18^F] FDDNP binds to both plaques (Aβ-aggregates) and tangles (tau-aggregates) for imaging (Small et al., 2006); [^18^F] AV-45 and [^11^C] PIB specifically bind to Aβ proteins for amyloid imaging. [^11^C] SB-13 and ^18^F-BAY94-9172 are also Aβ protein tracers (Ono et al., 2003; Verhoeff et al., 2004; Rowe et al., 2008). AV-1451 is a molecular probe that binds to tau protein (Devous Sr. et al., 2021). These imaging agents are used in the brain to reflect the progression and severity of AD pathology non-invasively through positron emission tomography (PET) scans, and have therefore become a more advanced and sensitive technique for the early diagnosis of AD, and can play an important role in monitoring outcomes. Among them, some specific molecular probes targeting Aβ and tau proteins have been approved by Food and Drug Administration of the United States (FDA) (Koole et al., 2009; Rinne et al., 2010; Snellman et al., 2014; Liu et al., 2015; Sabri et al., 2015; Devous Sr. et al., 2021). In particular, for [^18^F] AV-45, phase III clinical trials and other research results have confirmed that there is a significant correlation between [^18^F] AV-45 PET images and the results of evaluation of Aβ deposition obtained at autopsy; its *in vivo* safety is good, and no serious adverse drug reactions have been found (Martínez-Valle et al., 2015; de Vries et al., 2021). Fleisher et al. (2011) revealed that [^18^F] AV-45 can not only diagnose AD early, but also effectively distinguish between AD patients, patients with mild cognitive impairment (MCI), and healthy elderly people, with relatively high sensitivity and specificity. The radiochemical synthesis of [^18^F] AV-45 involves direct nucleophilic radiofluorination of the O-tosylated precursor in anhydrous DMSO at 115°C, followed by acid hydrolysis of the N-Boc protecting group and purification of the crude reaction mixture using solid phase extraction or high-performance liquid chromatography (HPLC). HPLC purification is costly, cumbersome and time-consuming to prepare. Solid phase extraction, by contrast, is simpler and more conducive to large-scale clinical applications (Choi et al., 2009; Liu et al., 2010; Zhang et al., 2020). Nevertheless, Compared to solid-phase extraction, higher purity products may be obtained by a preparative HPLC-equipped automated radiochemical synthesizer. Hank F. Kung’s team has studied the synthesis, influencing factors and purification of [^18^F] AV-45 on multiple levels (Choi et al., 2009; Liu et al., 2010; Zhang et al., 2020). The present experiments are based on their studies and adapt their synthesis and purification methods for use on Tracerlab FX_F-N_ synthesizer.

In order to provide colleagues with some useful references for the synthesis of [^18^F] AV-45, this paper describes the automated synthesis of [^18^F] AV-45 and the online purification of the crude product using preparative HPLC to obtain a high purity product.

## Materials

### Instruments and Consumables

The ^18^F^−^ used was produced using a MINItrace Qilin cyclotron (GE Healthcare, Uppsala, Sweden). Radiochemical synthesis and purification of [^18^F] AV-45 was carried out using a Tracerlab FX_F-N_ Synthesizer (GE Healthcare, United States). Sterilization of the final product was performed using a vented Millex-GS filter unit (Merck Millipore Ltd., Ireland). Analytical HPLC was performed on an Alltech 0201-0000 HPLC equipped with UV detector and radiation detector. The analytical HPLC column was an InertSustain C18 column (4.6 × 250 mm, 5 μm, Shimadzu-GL, Japan). The thin-layer chromatography plate (MACHEREY-NAGEL GmbH and Co.. KG, Germany) used with pretreated silica gel G plate, Pre-processing steps:Take 3 ml of 100 g/L chloroplatinic acid solution, add 97 ml of water and 100 ml of 60 g/L potassium iodide solution and mix well. Soak the silica gel G plate in the above solution for 5–10 s and dry for 12 h at room temperature, protected from light. The preparative HPLC column was an NUCLEOSIL 100-7 C18 column (16 × 250 mm, 7 μm, MACHEREY-NAGEL, Germany). Accurate pH test paper (pH range: 5.5–9.0, Q/GHSC 1571-2012) was purchased from SHANGHAI SSS REAGENT Co., Ltd. QMA cartridges (sep-pak light) and C18 cartridges (sep-pak plus) were purchased from Waters (Milford, MA, United States).

### Reagents and Solvents

All reagents and solvents were of analytical reagent grade. (E)-2-(2-(2-(2-Fluoroethoxy)ethoxy)ethoxy)-5-(4-methylaminostyryl)pyridine (AV-45 standards) (Lot 17061401) and (E)-2-(2-(2-((6-(4-((tert-butoxycarbonyl) (methyl)amino)styryl)-pyridin-3-yl)oxy)ethoxy)ethoxy)ethyl4-methylbenzenesulfonate (AV-45 precursors) were purchased from Wuxi Jiangyuan Industrial Technology and Trade Corporation. Acetonitrile, NaHCO_3_, kryptofix222 (K2.2.2), HCl, NaOH, DMSO, K_2_CO_3_, Ethanol, Saline for injection and water for injection were purchased from ABX (ABX Advanced Biochemical Compounds, Germany).

## Methods

### Optimization of Preparative HPLC Mobile Phase

The Preparative HPLC was configured with a NUCLEOSIL 100-7 C18 column with UV absorbance detection at 254 nm, a flow rate of 4.0 ml/min and a column temperature of 25°C. The concentration of AV-45 dissolved in acetonitrile was approximately 20 μg/ml and the injection volume was 2 ml. The mobile phases of 55:45, 65:35, 70:30, and 80:20 (V/V) acetonitrile-water were tested in order to identify the ideal retention time to peak, best peak shape for AV-45, and the separation from other substances.

### Optimization of Analytical HPLC Mobile Phase

The Analytical HPLC was equipped with an InertSustain C18 column with UV detection at 254 nm, a flow rate of 1.0 ml/min and a column temperature of 25°C. The concentration of AV-45 dissolved in acetonitrile was approximately 10 μg/ml and the injection volume was 25 μl.The mobile phases of (50:50, V/V; 60:40, V/V; and 70:30, V/V) acetonitrile-10 mmol/L ammonium formate were tested in order to identify the ideal retention time to peak, best peak shape of AV-45, and the separation from other substances.

### Automatic Radiochemical Synthesis of [^18^F] AV-45

#### Preparation Before Synthesis

1) Wash QMA cartridge with 10 ml of 0.5 M NaHCO_3_ solution, rinse with 20 ml of water for injection and blow it dry. Wash C18 cartridge with 10 ml of absolute ethanol, then rinse with 20 ml of water for injection and blow it dry.
2) Prepare 1.5 ml of K2.2.2–potassium carbonate solution (15 mg of K2.2.2 and 2.7 mg of K_2_CO_3_ were dissolved in 1.04 ml of acetonitrile and 0.46 ml of water), 0.25 ml of 3 M HCl solution, 3 ml of 1% NaOH solution, 5 ml of water and 1 ml of absolute ethanol.
3) Take out a bottle of AV-45 precursor (1 mg) and dissolve it with 1 ml of DMSO; have a bottle with 2 ml of anhydrous acetonitrile on standby.
4) The preparative HPLC and analytical HPLC were pre-equilibrated 1 h in advance.

#### Radiochemical Synthesis of [^18^F] AV-45

**SCHEME 1:**
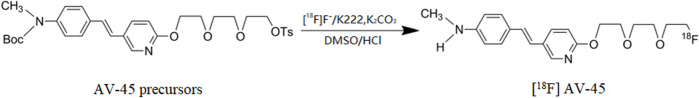
Radiosynthesis of [^18^F] AV45.

The radiochemical synthesis of [^18^F] AV-45 was carried out according to the reaction shown in Scheme 1. Radiolabelling was performed by heating 1 mg of O-tosylated precursor in anhydrous DMSO for 10 min at 115°C for nucleophilic radiofluorination, followed by hydrolysis of the N-Boc protecting group with 3 M HCl (0.35 ml), cooling to room temperature and neutralisation with 0.25 M NaOH (4.2 ml). The crude reaction mixture was then purified by injection on preparative HPLC. The fractions collected from the preparative HPLC were diluted with water (100 ml) and loaded onto a C18 cartridge. The pure [^18^F] AV-45 was enriched by C18 cartridge and eluted with 2 ml of anhydrous ethanol to obtain the [^18^F] AV-45 end product.

**FIGURE 1:**
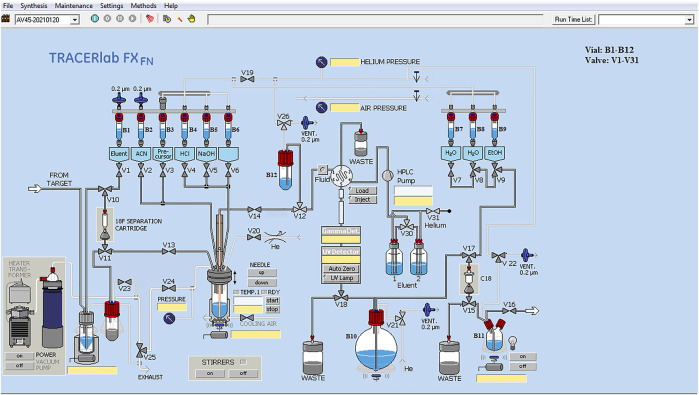
[^18^F] AV-45 synthesis software operation interface.

Automatic radiochemical synthesis of [^18^F] AV-45 was carried out on a Tracerlab FX_F-N_ synthesizer (Figure 1). Before starting the synthesis, we added all reagents to their corresponding test tubes as follows: B1, 1.5 ml K2.2.2-K_2_CO_3_ solution; B2, 2 ml of anhydrous acetonitrile; B3, 1 mg of AV-45 precursor dissolved in 1 ml of anhydrous DMSO; B4, 0.35 ml of 3 M HCl solution; B5, 4.2 ml of 0.25 M NaOH solution; B7, 15 ml of H_2_O; B8, 15 ml of H_2_O; B9, 2 ml of absolute ethanol; B10, 100 ml of water; B11, 11 ml of 0.9% normal saline.

The synthesis process was automatically controlled by software in a shielded hot chamber. The synthesis steps were as follows: [^18^F] ions (33.3–37 GBq) were captured by QMA cartridge. Then, the active fluorine ions were eluted into the reaction flask through 1.5 ml of a K2.2.2-K_2_CO_3_ solution. The mixed solution was stirred in a reaction flask and reacted at 110°C under negative pressure and helium flow for 5 min, then the reaction flask was cooled to 75°C. Then 2 ml of anhydrous acetonitrile was added to the residue, azeotropically dried under vacuum, then under helium flow at 110°C for 5 min and cooled to 35°C. The precursor solution was added to the reaction flask and airtight heating to 115°C for 10 min, then the reaction mixture was cooled to 40°C. At this time, 0.35 ml of 3 M HCl solution was added to the reaction mixture and reacted at 115°C for 5 min, before the reaction flask was cooled to 35°C. Then 4.2 ml of 0.25 M NaOH solution was added to the mixture for neutralization. The [^18^F] AV-45 mixture was then transferred to B12 flask in preparation for the HPLC injection.

The preparative HPLC flow rate was set as 4 ml/min, the mobile phase was acetonitrile-water (60:40, V/V), and the chromatographic column was a preparative C18 reversed-phase chromatographic column. The purified [^18^F] AV-45 were collected into a round-bottom flask (B10), then the samples were transferred from B10 to the C18 cartridge for loading. Subsequently, the C18 cartridge was washed with 15 ml of deionized water from the B7 flask and a further 15 ml of deionized water from the B8 flask. The cartridge was dried under a stream of helium, then pure [^18^F] AV-45 was eluted with 2 ml of ethanol (B9) into the transfer flask (B11), to which 11 ml of 0.9% normal saline was added in advance. The final product passed through the 0.22 μm sterile filter and was collected into sterile vials to obtain products for injection.

### Quality Control

The chemical purity and specific activity of [^18^F] AV-45 were determined by HPLC and quantified using the calibration curve of an F-19 standard. Radiochemical purity was determined by HPLC. The residual K2.2.2 limit was tested by thin-layer chromatography. The final product was used as the test article, and the precisely prepared control solution (50 μg/ml), 2.5 μl of which was taken and dotted on the pretreated silica gel G thin-layer chromatography plate at the same time, was checked 1 min later. Compared with water, the center of the spot showed a dark blue circle or ring. If the spot center of the test solution exhibits a dark blue color, it should be lighter than the dark blue in the center of the control solution. A small volume (50 μl) of the final product was dropped on the precision pH test strip (pH range: 5.5–9.0) and the pH value of the product was determined by comparing it with the standard color card provided by the test paper manufacturer. Sterility (direct inoculation method) and bacterial endotoxin tests (gel method) were performed after decay (testing laboratory: The Center for Medical Laboratory, the First Teaching Hospital of Xinjiang Medical University, No.1. Liyushan Road, Urumqi, Xinjiang, China).

## Results

### Optimization of Preparative HPLC Mobile Phase Ratio

**FIGURE 2:**
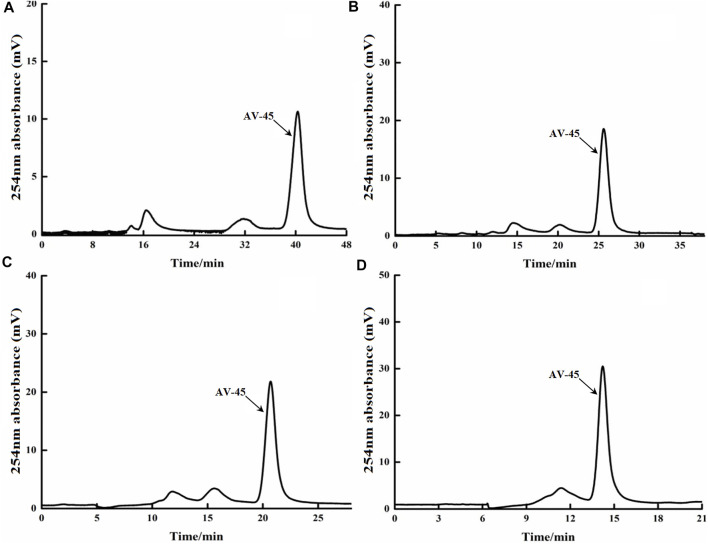
Retention times of AV-45 in preparation HPLC column with different mobile phase ratios. The arrow points to the AV-45 standard peak.

The HPLC preparation program, edited separately by Tracerlab FX_F-N_, was run; the deuterium lamp and the preparation HPLC column were pre-equilibrated for about 40 min, then loading was started. Approximately 20 μg/ml of AV-45 standard solution was loaded, with each injection volume being 2 ml. The mobile phases of acetonitrile-water (55:45, 65:35, 70:30, and 80:20, all V/V) were tested to determine the ideal retention time to peak and peak shape of AV-45. The results are presented in Figure 2.

When the mobile phase ratio was acetonitrile-water (55:45, V/V), the time to peak of the AV-45 standard was approximately 40 min (Figure 2A); when the mobile phase ratio was acetonitrile-water (65:35, V/V), the time to peak of the AV-45 standard was approximately 24 min (Figure 2B); when the mobile phase ratio was acetonitrile-water (70:30, V/V), the time to peak of the AV-45 standard was approximately 20 min (Figure 2C); when the mobile phase ratio was acetonitrile-water (80:20, V/V), the time to peak of the AV-45 standard was approximately 15 min (Figure 2D). Having considered the progress and preparation time of the synthesis test, the configuration scheme with a mobile phase ratio of acetonitrile-water (65:35, V/V) was selected for this experiment.

### Optimization of Analytical HPLC Mobile Phase Ratio

**FIGURE 3:**
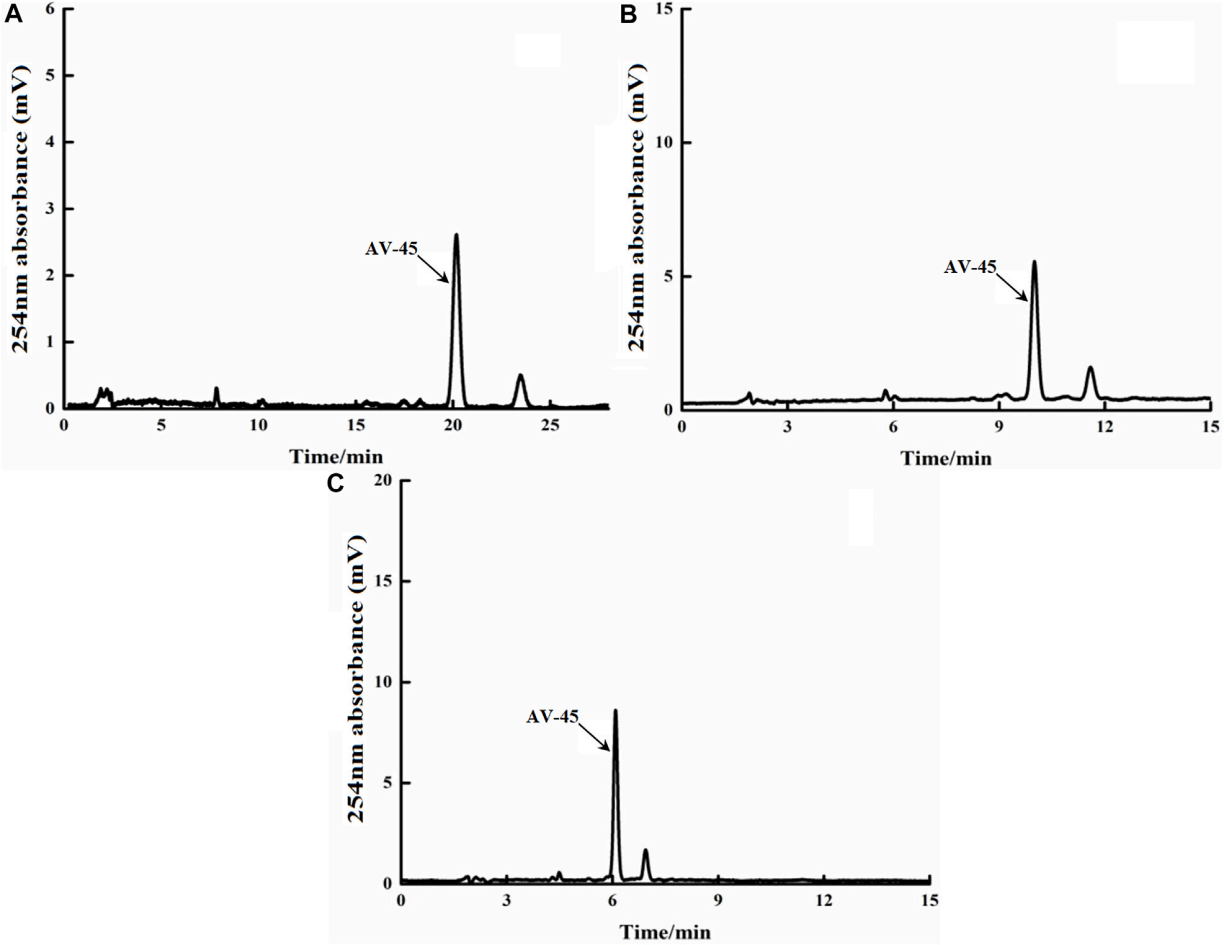
Retention times of AV-45 in analytical HPLC column with different mobile phase ratios. The arrow points to the AV-45 standard peak.

The Altech analytical HPLC preparation program was run, the deuterium lamp and the preparation HPLC column were pre-equilibrated for about 30 min, and then loading was started. Approximately 10 μg/ml of AV-45 standard solution was loaded, with each injection volume being 25 μl. The mobile phases of acetonitrile-10 mmol/L ammonium formate (50:50, 60:40, and 70:30, all V/V) were tested to determine the ideal retention time to peak of AV-45, better peak shape of AV-45, and separation from other substances. The results are presented in Figure 3.

When the mobile phase ratio was acetonitrile-10 mmol/L ammonium formate (50:50, V/V), the time to peak of the AV-45 standard was about 20.1 min (Figure 3A); when the mobile phase ratio was acetonitrile-10 mmol/L ammonium formate (60:40, V/V), the time to peak of the AV-45 standard was about 9.8 min (Figure 3B); when the mobile phase ratio was acetonitrile-10 mmol/L ammonium formate (70:30, V/V), the time to peak of the AV-45 standard was about 6.1 min (Figure 3C). Based on the analysis time and peak stability, the configuration scheme with a mobile phase ratio of acetonitrile-10 mmol/L ammonium formate (60:40, V/V) was selected for this experiment.

### Applicability Test of Analytical HPLC System

**FIGURE 4:**
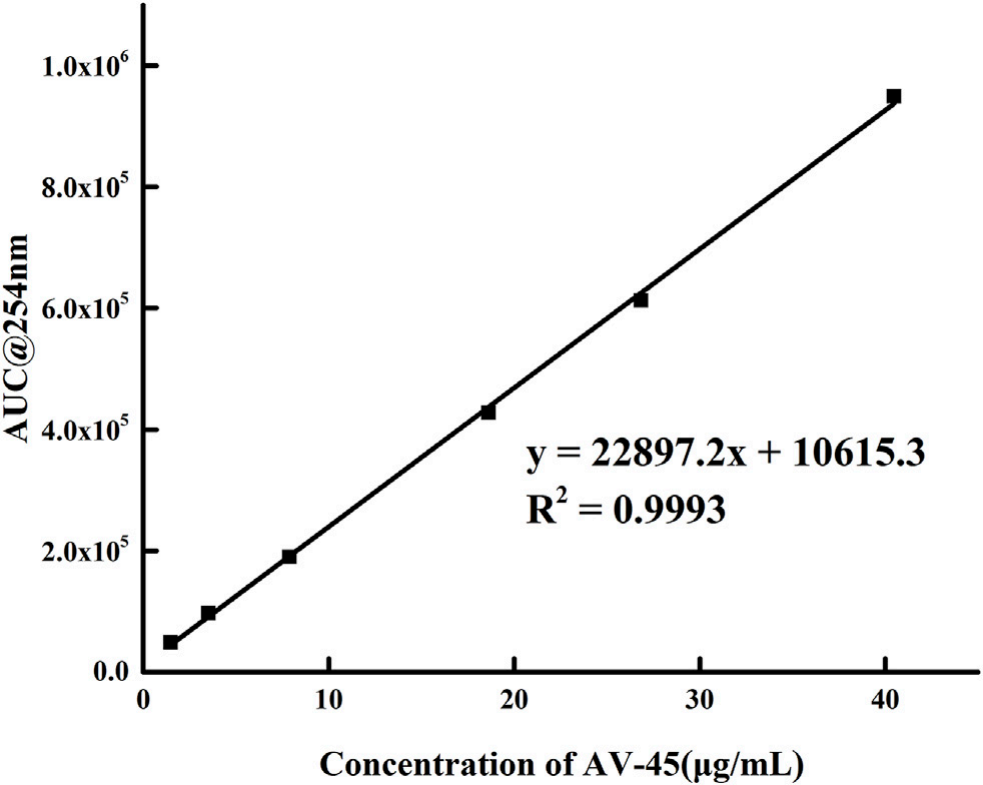
Working curve of AV-45 series concentration solution.

Series concentration solutions of the AV-45 standard were prepared, the HPLC flow rate was set to 1.0 ml/min, the mobile phase ratio was the configuration scheme with acetonitrile-10 mmol/L ammonium formate (60:40, V/V), and the absorption peak areas of a series of solutions were measured at 254 nm on the analytical HPLC. Within the concentration range of 1.47–40.48 μg/ml, the linear equation was given by *y* = 22,897*x* + 10,615 and the correlation coefficient was *r* = 0.9996 (*n* = 6). The concentration of the AV-45 standard had a clear linear relationship with the absorption peak area (Figure 4). The detection limit was 0.51 μg/ml, the limit of quantitation was 1.53 μg/ml, the accuracy was 1.02% (*n* = 9), the repeatability was 0.79% (*n* = 9), and the intra- and inter-day precision (RSD%) were 0.92 and 1.6.

### Automatic Radiochemical Synthesis of [^18^F] AV-45

**FIGURE 5:**
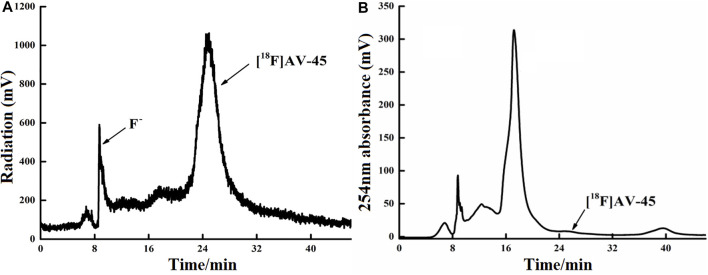
Curve of [^18^F] AV-45 in preparative HPLC during radiochemical synthesis. **(A)** The samples curve obtained by the radioactivity detector, in which the first peak is the F^−^ peak, and the second, larger peak is the [^18^F] AV-45 peak; the time to peak of [^18^F] AV-45 is about 24 min, similar to the AV-45 standard. **(B)** The samples curve obtained by the ultraviolet detector, and the [^18^F] AV-45 peak, indicated by the arrow, is very small.

**FIGURE 6:**
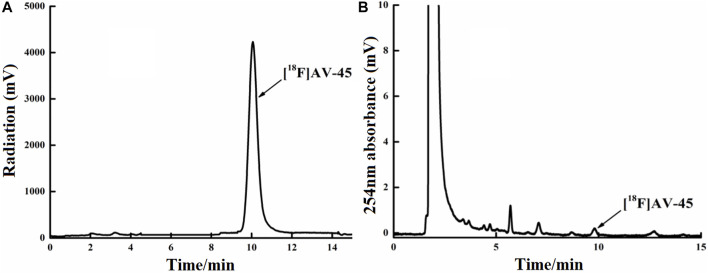
Curve of the [^18^F] AV-45 final product in analytical HPLC (undiluted). **(A)** The samples curve obtained from the radioactivity detector, in which the only large peak is the [^18^F] AV-45 peak; the time to peak of [^18^F] AV-45 is approximately 9.8 min, similar to the AV-45 standard. **(B)** The samples curve obtained by the ultraviolet detector, in which the first, larger peak is the ethanol peak, and the [^18^F] AV-45 peak, indicated by the arrow, is very small; this corresponds to the radioactive peak.

The automatic radiochemical synthesis of [^18^F] AV-45 was carried out through a synthesis program we edited ourselves (Tracerlab FX_F-N_, Figure 1). Four repeats of production were carried out at different times, all of which were successful. The yields of the preparations were increased (^18^F^−^ activity was 33.3–37 GBq), the average non-decay corrected radiochemical yield of [^18^F]AV-45 was 14.8 ± 2.1% (*n* = 4), and the total synthesis time averaged 60 ± 5 min. Quality control was carried out on the four batches of products, and the results were consistent with the guiding principles for the quality control of positron emitting radioactive drugs. At the end of synthesis, analytical HPLC radioactivity detection analysis demonstrated that the radiochemical purity of [^18^F] AV-45 was >95% and the specific activity was 72.9 ± 10.2 MBq/μg. The chemical properties were confirmed by analytical HPLC in comparison with a non-radioactive AV-45 standard; the product and the AV-45 standard showed the same retention time under the HPLC conditions described in *Optimization of Preparative HPLC Mobile Phase Ratio–Optimization of Analytical HPLC Mobile Phase Ratio* (Figures 5, 6). The main quality control items required for radioactive drug injection, including physical properties, pH value, K2.2.2 residual, Chemical impurities (concentration less than 5 μg/ml), etc., met the requirements (Table 1). The average yield of a single synthesis of [^18^F]AV-45 is 4.9 ± 0.7 GBq, and based on 370 Mbq per person per image, a single production run can be used for 5-8 people for diagnostic imaging.

**TABLE 1:** Quality control results of four batches of [^18^F] AV-45 (*n* = 4).

Parameter	Value	Parameter	Value
Physical characteristics	Clear, colorless liquid; no suspended particles	Synthesis time	60 ± 5 min
pH value	6.5	Radiochemical yield	14.8 ± 2.1%
Radiochemical purity	97.3 ± 0.5%	Content of K2.2.2	<50 μg/ml
Sterile	Yes	Specific activity	72.9 ± 10.2 MBq/μg
Bacterial endotoxin (IU)	<2.0	Chemical quantity	5.21 ± 0.26 μg/ml
Activity concentration	380.0 ± 52.9 MBq/ml	Yield of [^18^F] AV-45	4.9 ± 0.7 GBq

## Discussion

In this study, we edited a synthesis and HPLC separation program based on the Tracerlab FX_F-N_ software platform, allowing the entire process to be automated. However, due to the lack of an automatic liquid flow detection system, the connection between the synthesis and purification procedures was not fluent, resulting in too-long waiting and confirmed loading times. There was a waiting time for confirmation between each step of synthesis. The time from the beginning of synthesis to obtaining the final product was about 60 min, which is unfortunately long (Yao et al., 2010; Hayashi et al., 2013; Li et al., 2016).

In this study, protective groups were hydrolyzed using acid, with the marked reaction temperature 115°C and the marked time 10 min. The HPLC purification method was adopted, a slight divergence from other reported methods, which may be the reason for the difference in synthetic efficiency. It can be seen from the UV absorbance and radioactivity analytical HPLC chromatograms, and the quality control results of the final product that the preparation obtained in this experiment can meet the requirements for clinical intravenous medication. The quality of this product is similar to that synthesized by other peers (Li et al., 2016).

**FIGURE 7:**
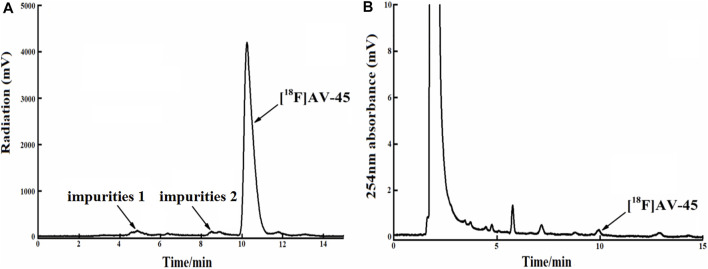
The radiochemical purity of the preparation is 92.6% when the samples are collected until 1/4 of the peak height in the tailing edge of the radioactive peak. **(A)** The analytical HPLC radioactivity spectrum of final product; Arrows mark the radioactive impurities and the [^18^F] AV-45 peak. **(B)** The analytical HPLC UV spectrum of the final product. The arrow points to the [^18^F] AV-45 peak.

**FIGURE 8:**
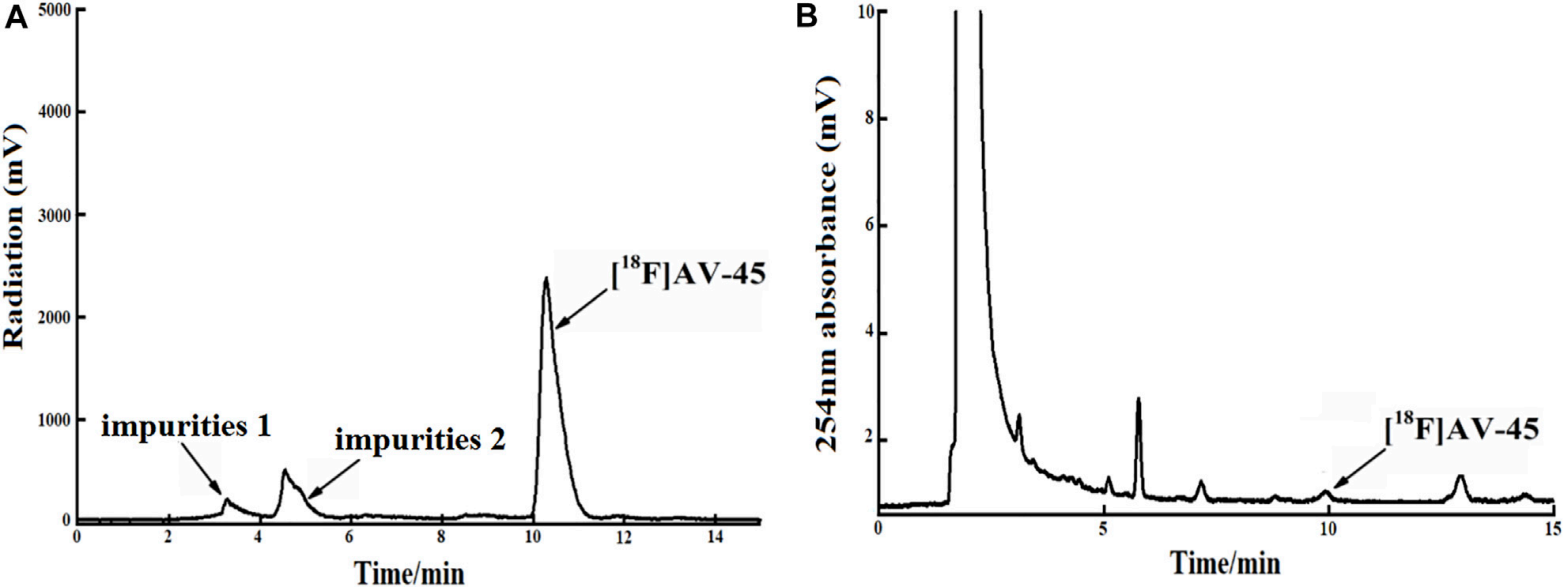
The radiochemical purity of the preparation is 78.9% when the samples are collected until 1/6 of the peak height in the tailing edge of the radioactive peak. **(A)** The analytical HPLC radioactivity spectrum of final product; Arrows mark the radioactive impurities and the [^18^F] AV-45 peak. **(B)** The analytical HPLC UV spectrum of the final product. The arrow points to the [^18^F] AV-45 peak.

In each production of [^18^F] AV-45 preparation, the [^18^F] AV-45 radioactive peak position (preparative HPLC) served as the reference time for samples collection (about 24 min), and the starting position of the radioactive peak served as the start of samples collection. It should be noted that, during collection of the preparative HPLC [^18^F] AV-45 samples, the radiochemical purity of the [^18^F] AV-45 preparation will be greater than 95% when the sample is collected with a cutoff near about 1/3 of the peak height at the trailing edge of the radioactive peak (Figure 6A shows that the radiochemical purity of the preparation was 98.5% when collecting samples until 1/3 of the peak height in the tailing edge of peak). when collecting samples in the range of 1/3–1/4 peak height in the tailing edge of the radioactive peak the proportion of radioactive impurities in the [^18^F] AV-45 preparation began to increase; however, the radiochemical purity of the preparation could still reach greater than 90% (Figure 7A shows that the radiochemical purity of the preparation was 92.6% when collecting samples until 1/4 of the peak height in the tailing edge of peak). When collecting samples in the range of less than 1/4 peak height in the tailing edge of the radioactive peak, the proportion of radioactive impurities in the [^18^F] AV-45 preparation will exceed the limit and the radiochemical purity of the preparation will be less than 90%. Therefore, the radiochemical purity of the whole preparation will be lower when the samples are collected at a position lower than 1/4 of the peak height (as shown in Figure 8A, the radiochemical purity of the preparation was 78.9% when collecting samples until 1/6 of the peak height in the tailing edge of peak). As demonstrated by comparison between Figures 7B, 8B, the samples of the [^18^F] AV-45 preparation collected until 1/6 of the peak height showed a slight increase in content of chemical impurities compared to those collected until 1/4 of the peak height, but no new chemical impurities appeared. Therefore, when collecting the preparative HPLC [^18^F] AV-45 samples, samples collection should be limited to the range of 1/3–1/4 peak height in the tailing edge of the radioactive peak. In addition, in this study, the separation of [^18^F] AV-45 from the impurities was not ideal during the crude product purification stage using the preparative HPLC (Figure 5B), and the sample volume could be reduced by concentrating the crude product to reduce the solvent content. The water content of the crude product was also adjusted to improve the peak shape, resulting in a more chemically pure product.

In the synthesis of [^18^F] AV-45 from the precursor, there are three main by-products, namely the substitution of the ^18^F substituent position by the chlorine atom, the OTs group and the hydroxyl group respectively. The hydroxyl derivative was the most abundant, and its UV absorption and retention time were closest to that of [^18^F] AV-45 (Zhang et al., 2005; Wei et al., 2007; Choi et al., 2009; Zhang et al., 2020). The other three major minor peaks appearing in Figure 6B may be the three aforementioned by-products, but the sum of the impurities is within the acceptable range for humans.

## Conclusion

In the present experiment, a [^18^F] AV-45 synthesis program and preparative HPLC separation program we edited ourselves were used for the synthesis and purification of [^18^F] AV-45 on a Tracerlab FX_F-N_. Products with high radiochemical and chemical purity were obtained, and the final product met the major requirements of radiopharmaceutical quality control. In the present experiment, the total synthesis time was too long, reaching 60 ± 5 min, and the synthetic yield was low (14.8 ± 2.1%), demonstrating the need for further optimization. Because the preparative HPLC was used for impurity separation, the final product obtained had high chemical purity, and almost no other impurities with high content were found in the analytical HPLC.

## Data Availability

The original contributions presented in the study are included in the article/Supplementary Material, further inquiries can be directed to the corresponding author.
